# Exposure to family planning messages and modern contraceptive use among men in urban Kenya, Nigeria, and Senegal: a cross-sectional study

**DOI:** 10.1186/s12978-015-0056-1

**Published:** 2015-07-22

**Authors:** Chinelo C. Okigbo, Ilene S. Speizer, Meghan Corroon, Abdou Gueye

**Affiliations:** Department of Maternal and Child Health, Gillings School of Global Public Health, University of North Carolina at Chapel Hill, Chapel Hill, NC USA; Measurement, Learning & Evaluation Project, Carolina Population Center, University of North Carolina at Chapel Hill, Chapel Hill, NC USA; Measurement, Learning & Evaluation Project, IntraHealth International, Dakar, Senegal

**Keywords:** Sub-Saharan Africa, Modern contraception, Men, Family planning programs, Urban

## Abstract

**Background:**

Family planning (FP) researchers and policy makers have often overlooked the importance of involving men in couples’ fertility choices and contraception, despite the fact that male involvement is a vital factor in sexual and reproductive health programming. This study aimed to assess whether men’s exposure to FP demand-generation activities is associated with their reported use of modern contraceptive methods.

**Methods:**

We used evaluation data from the Measurement, Learning & Evaluation project for the Urban Reproductive Health Initiative (URHI) in select cities of three African countries (Kenya, Nigeria, and Senegal) collected in 2012/2013. A two-stage cluster sampling design was used to select a representative sample of men in the study sites. The sample for this study includes men aged 15–59 years who had no missing data on any of the key variables: 696 men in Kenya, 2311 in Nigeria, and 1613 in Senegal. We conducted descriptive analyses and multivariate logistic regression analyses to assess the associations of interest. All analyses were weighted to account for the study design and non-response rates using Stata version 13.

**Results:**

The proportion of men who reported use of modern contraceptive methods was 58 % in Kenya, 43 % in Nigeria, and 27 % in Senegal. About 80 % were exposed to at least one URHI demand-generation activity in each country. Certain URHI demand-generation activities were significantly associated with men’s reported use of modern contraception. In Kenya, those who participated in URHI-led community events had four times higher odds of reporting use of modern methods (aOR: 3.70; *p* < 0.05) while in Senegal, exposure to URHI-television programs (aOR: 1.40; *p* < 0.05) and having heard a religious leader speak favorably about FP (aOR: 1.72; *p* < 0.05) were associated with modern contraceptive method use. No such associations were observed in Nigeria.

**Conclusion:**

Study findings are important for informing future FP program activities that seek to engage men. Program activities should be tailored by geographic context as results from this study indicate city and country-level variations. These types of gender-comprehensive and context-specific programs are likely to be the most successful at reducing unmet need for FP.

**Electronic supplementary material:**

The online version of this article (doi:10.1186/s12978-015-0056-1) contains supplementary material, which is available to authorized users.

## Background

Family planning (FP) has many societal benefits including reduction of maternal and infant mortality, improved economic development through increased women’s participation in the labor force, and more sustainable use of resources due to reduced population growth [1–4]. However, many women do not practice FP despite their desire to delay or avoid childbearing [1]. It is estimated that a quarter of women of reproductive age in the developing world want to avoid pregnancy but are not using an effective contraceptive method (in other words, they have an unmet need for FP) [5]. This translates to an estimated 225 million women in developing countries having an unmet need for modern contraception [5]. The burden is worse in sub-Saharan Africa where it is estimated that, in 2012 alone, 60 % of women of reproductive age (i.e. ages 15–49) had an unmet need for modern contraception [6]. According to the most recent Demographic and Health Surveys (DHS) in Kenya (2014), Nigeria (2013), and Senegal (2010/11), the proportions of women in union (i.e. married or cohabiting with a male partner) using a modern contraceptive method are 53, 10, and 12 % respectively [7–9]. These low levels of modern contraceptive prevalence are discouraging, especially in light of the region’s high fertility and high maternal and infant mortality rates. With such low levels of modern contraceptive method use, it is expected that the unmet need for FP will be high. However, only 18, 16, and 30 % of women in union reported having an unmet need for FP in Kenya, Nigeria, and Senegal, respectively [7, 8, 9]. These low proportions of unmet need for FP indicate that high fertility desires continue to persist in these countries.

Several studies have found that women in sub-Saharan Africa face many sociocultural barriers to use of modern contraceptive methods [10–12]. A common reason for non-use of modern methods is male partner opposition to FP [12–18]. A number of studies show that men’s reproductive intentions (or women’s perceptions of men’s intentions) affect the contraceptive behavior of their female partners [13, 17, 19, 20]. This is often manifested through men’s disapproval of FP, their desire for more children, and/or lack of spousal communication about FP. Despite men’s key role in sex and reproduction, they are often left out of FP discourses and programs.

Two decades have passed since the 1994 International Conference on Population and Development (ICPD) that led to recommendations to involve men as clients, partners, and agents of change in sexual and reproductive health, including FP [21]. Male involvement was positioned as essential to the success of FP programs [21–23]. However, not many FP programs have involved men. Among the few studies in sub-Saharan Africa that include men, a majority focus on factors associated with couples’ FP practices within the context of HIV/AIDS [15, 23, 24] or men’s reported knowledge and use of contraceptive methods [25–28] rather than the impact of reproductive health programs on men’s contraceptive behaviors. Therefore, there remains a dearth of knowledge on the best strategies to engage men in sexual and reproductive health programs and the impact of male involvement on FP adoption and continuation. Scientific evidence exists on the impact of FP programs on increasing contraceptive ideation/approval and use, and reducing fertility among women [29–34]. However, less is known about the impact of such programs on men’s involvement in couples’ use of modern contraceptive methods.

One evaluation study that included married men in northern Ghana found that men who reported being encouraged to use FP by at least one person in their social network were more likely to approve of FP and to discuss contraceptive use with their wives compared to those who did not receive such encouragement [35]. Men’s increased FP approval and communication were found to be associated with their female partners’ increased use of modern contraceptive methods [36]. A second study examined a peer-delivered educational FP intervention that targeted married men in Malawi and found increased uptake of modern contraceptive methods (especially male condoms and injections) through improved spousal communication about FP [37]. A third study, conducted among men in rural Gambia, showed that involving religious leaders in teaching about the connection between Islam, health, and FP resulted in increased knowledge of the different contraceptive methods and a 13 percentage-point increase in couples’ contraceptive use over a one year period [38]. Further, Terefe and Laron (1993) conducted a field trial in a semi-urban community in Ethiopia on the efficacy of home visitation by health personnel on women’s modern contraceptive use, with or without the husbands’ participation [39]. They found that involving the husbands during the home visits resulted in increased uptake of contraceptive methods by couples two months following the intervention and was sustained even after 12 months. In addition, there was less discontinuation of the contraceptive methods in the experimental group (husband participation) compared to the control group (wife only) [38]. Lastly, a 2003 study conducted in Uganda examined the relationship between exposure to behavior change communication (BCC) campaigns in the media (television, radio, posters, and print media) and use of modern contraceptive methods among men and found a positive dose-response effect between media exposure to FP messages and modern contraceptive use, mostly male condoms [40]. In summary, targeted FP messages delivered by peer educators, health personnel, religious leaders, and multimedia sources seem to have positive associations with men’s FP use and, in some cases, couples’ use of modern contraceptive methods. Many of these studies involved the evaluation of a single-strategy FP program. Thus, little is known about the effect of a multi-strategy FP intervention that includes community education and media campaigns. Implementing a multi-strategy FP intervention may reach a broader audience and possibly have a larger impact on modern contraceptive use.

FP programs may also have heterogeneous effects on pregnancy prevention depending on the geographic context, for example rural versus urban contexts. The high rate of urbanization coupled with little or no change in urban development, especially in sub-Saharan Africa, has led to concentrated poverty in urban areas resulting in an increased number of slums [11]. The health and development challenges faced in urban slums are likely different from those faced in poor rural areas. Hence, there is a need to understand the effect of FP programs in urban areas especially in those areas with high poverty and fertility rates. Our study helps to fill the gap in information on the effect of a FP program on use of modern contraceptive methods among men in select urban areas in sub-Saharan Africa. We assessed the association between exposure to a multi-strategy FP program implemented in select urban areas in Kenya, Nigeria, and Senegal and the report of modern contraceptive method use by men in the studied areas. Identifying strategies to increase men’s approval and use of modern contraception is relevant to improving FP practice in sub-Saharan Africa.

## Methods

### The urban reproductive health initiative

In 2009, the Bill & Melinda Gates Foundation launched the Urban Reproductive Health Initiative (URHI), in four countries (India, Kenya, Nigeria, and Senegal) with the goal of increasing contraceptive prevalence rates in selected cities. The URHI was locally-designed to address factors that affect FP supply, demand, and advocacy in each country. The Measurement, Learning & Evaluation (MLE) project is the independent evaluation arm of the URHI and uses a rigorous study design to assess the impact of the country-specific URHI on FP use in project cities in each country. Information on several sexual and reproductive health (SRH) and maternal and child health (MCH) indicators was collected at the household level from eligible men and women of reproductive ages. Baseline data were collected to provide pre-program information while midterm data (two years after baseline) were collected to provide information on the effect of the program on select SRH/MCH program indicators and to inform mid-course corrections. The focus of the midterm data collection was on the demand-generation activities; these activities varied across the countries but generally aimed to improve knowledge of contraceptive methods, foster FP discussion, and increase social approval for FP. More information about the country-specific URHI and the MLE project are detailed on the MLE project website [41]. Here, we focus on the midterm evaluation data from men in the three African countries. We assessed six demand-generation activities of the Nigerian Urban Reproductive Health Initiative (NURHI), five demand-generation activities undertaken by the Kenya URHI program, termed *Tupange* (Swahili for “Let’s Plan”), and five demand-generation activities of the Senegal URHI, termed l’Initiative Sénégalaise de Santé Urbaine (ISSU) in French. The demand-generation activities include exposure to FP messages via mass media, print media, interpersonal communication, and community events as listed in Table 1. Details of the intervention programs and the evaluation of the impact of these activities on women’s use of modern contraceptive methods are presented elsewhere [31]. The main objective of this study is to assess the association between the country-specific demand-generation program activities and use of modern contraceptive methods as reported by men in the three African countries.

**Table 1:** Urban Reproductive Health Initiative demand-generation activities in the three countries

URHI demand generation program activities: In the past year,	Kenya	Nigeria	Senegal
	Tupange	NURHI	ISSU
Listened to any URHI radio programs	✓	✓	✓
Saw any URHI television programs	✓	✓	✓
Participated in any URHI community events	✓	✓	✓
Exposed to any URHI print media^α^	✓	–	–
Exposed to any URHI logos/brands	✓	✓	–
Heard/saw any URHI English slogans	–	✓	–
Heard/saw any URHI local language slogans	–	✓	–
Heard a religious leader speak in favor of family planning	–	–	✓
Heard at least one URHI radio advert on family planning	–	–	✓
Total number of demand generation program activities	5	6	5

*URHI* Urban Reproductive Health Initiative, *NURHI* Nigerian Urban Reproductive Health Initiative, *ISSU* l’Initiative Sénégalaise de Santé Urbaine

^α^Tupange program print media includes newspaper, magazine, comic books, posters, leaflets, and brochures

### Study design and sample

The MLE project uses a robust study design and multiple data collection strategies to gather information needed to evaluate the impact of the URHI in each country. The household-based surveys collected data from a longitudinal sample of women and from repeated cross-sectional samples of men from select cities during each wave of data collection. This study uses midterm evaluation data collected from a cross-sectional sample of men in 2012 in Kenya (Mombasa), Nigeria (Ibadan and Kaduna), and in 2013 in Senegal (Guédiawaye, Pikine, and Mbao).

In each city, a two-stage cluster sampling design was used to select a representative sample of men and women to be included in the baseline survey. The first stage included a selection of primary sampling units (PSUs) or clusters while the second stage included a selection of households within each PSU. All eligible men and women in the selected households were surveyed at baseline. At midterm, the baseline women were followed longitudinally while a new cross-sectional sample of men was obtained. Because the men’s and women’s surveys were conducted at the same time, the same PSUs from the baseline survey were used. The households within these PSUs were re-listed and mapped prior to the midterm survey. A systematic random sample of households was then selected for inclusion in the midterm survey. In Kenya, where only one city (Mombasa) was surveyed, a total of 76 PSUs were included and in each PSU, 15 households were selected for inclusion in the men’s cross-sectional survey. In Nigeria, 56 PSUs were selected in each of the two cities (Ibadan and Kaduna); in each selected PSU, 20 households were randomly selected for the men’s survey. Three cities were included for the midterm men’s survey in Senegal. Thirty-one PSUs were selected in each of the three cities (Guédiawaye, Pikine, and Mbao) and 11 households per PSU were selected. The differing numbers of PSU and households selected per PSU in each country/city were calculated based on our key indicators of interest (primarily contraceptive prevalence rate) and information from the most recent country-specific DHS on the number of eligible men (and women) identified per household, response rates, and DHS sampling strategies by country. In all three countries, all men aged 15–59 from the selected households were eligible to participate in the study. Upon receipt of informed consent, eligible men were interviewed by a male interviewer using a paper-and-pencil questionnaire in a private location within or close to the households. The response rates were 74 % in Kenya, 96 % in Nigeria, and 85 % in Senegal giving sample sizes of 696, 2358, and 1622 men respectively [42–44]. For this analysis, our sample was restricted to 696 men in Kenya, 2311 men in Nigeria, and 1613 men in Senegal who had no missing data on any of the key variables.

### Measures

The outcome variable is the use of modern contraceptive methods, defined as the man’s report of whether he or his partner was using any of the following methods at the time of interview: male condom, female condom, male sterilization, female sterilization, daily pills, injection, implant, intrauterine device (IUD), emergency pills, diaphragm, spermicides (gels and foams), lactational amenorrhea method (LAM), and standard days method (SDM). The outcome variable was coded as ‘1’ if the man answered ‘yes’ to any of the modern methods and ‘0’ otherwise. The key exposure variables include the country-specific demand-generation activities (see Table 1) such as exposure to FP messages via radio and television programming and advertisements, print media, program branding (logo) materials, community outreach events, and engagement of religious leaders. The respondents were asked if they had been exposed to each of the program demand-generation activities in the year prior to survey. To assess the association between the intensity of program exposure and modern contraceptive method use, we created an index score that measured the number of demand-generation activities each respondent was exposed to in each of the countries. The index score ranged from zero (i.e. no program exposure) to five (exposed to all program activities in Kenya and Senegal) or six (exposed to all program activities in Nigeria). The association between this intensity of program exposure variable and the outcome measure was assessed.

We controlled for factors known to be associated with modern contraceptive method use. These control variables included sociodemographic factors such as the respondents’ age, educational attainment, marital status, religious affiliation, household wealth index, and city of residence. The household wealth index was created as in standard Demographic and Health Surveys [45]. The categorization of these control variables is shown in Table 2.

**Table 2:** Sociodemographic characteristics of men aged 15–59 in the three countries

Characteristics	Kenya (%)	Nigeria (%)	Senegal (%)
Age			
15–24	27.5	26.8	31.8
25–34	34.3	30.6	31.8
35–44	21.9	22.9	21.1
45–59	16.3	19.7	15.3
Education			
Primary or less	42.0	14.9	55.7
Secondary	37.3	53.5	36.1
Higher	20.7	31.6	8.2
Marital status			
Single/divorced/widowed	39.7	42.6	56.3
Married/living together	60.3	57.4	43.7
Religion			
Christian/Others	64.7	47.0	5.4
Muslim	35.3	53.0	94.6
Wealth index			
Poorest	21.3	20.0	25.0
Poor	22.7	19.8	18.9
Middle	21.3	20.0	19.0
Rich	17.7	20.5	18.3
Richest	17.0	19.7	18.8
City of residence (Nigeria/Senegal)			
Ibadan / Guédiawaye	–	52.4	32.1
Kaduna / Pikine	–	47.6	33.0
NA / Mbao	–	–	34.9
Weighted N	696	2311	1613

All analyses are weighted (across-city weights were used in Nigeria and Senegal)

### Analysis

All analyses were weighted based on probability of selection, response rates, and adjusted for the clustered survey design. This was done using the ‘*svy*’ function in Stata version 13 [46]. Descriptive analyses were conducted and logistic regression analyses were performed to assess the single and cumulative associations between the demand-generation activities and modern contraceptive method use. All models were run separately for each of the three countries. City-level analyses were run for the two cities in Nigeria and the three cities in Senegal; however, the city-level results are only available as additional files. The goodness-of-fit tests for all models were assessed and results indicated that the final models fit the data appropriately. Ethical approval for the study protocol and the informed consent process was obtained from the Institutional Review Board at the University of North Carolina at Chapel Hill and the Ethical Review Committee Boards in Kenya, Nigeria, and Senegal.

## Results

### Sociodemographic characteristics

Table 2 shows the sociodemographic characteristics of men by country. About 52 % of men surveyed in Nigeria reside in Ibadan, which is the capital city of Oyo State in southwestern Nigeria while the rest reside in Kaduna, the capital city of Kaduna State in northwestern Nigeria. In Senegal, about a third of the men reside in each of the three cities included: Guédiawaye, Mbao, and Pikine located in Dakar Senegal. All men in the Kenya sample are from Mombasa. Across the three countries, the analysis samples were young with more men in the 15–24 and 25–34 age groups. The men in Nigeria are more educated as only about 15 % had primary, Quranic, or no formal education compared to 42 % in Kenya and 56 % in Senegal. Approximately 60 % of the Kenya sample was married or cohabiting with their partner compared to 57 % in the Nigeria sample and 44 % in the Senegal sample. The highest proportion of men who were Muslims was found in Senegal (95 %) followed by Nigeria (53 %), and then Kenya (35 %). Across all countries, the household wealth indices were calculated and divided into quintiles; so it is expected that about 20 % of the sample would fall into each of the five categories. See Additional file 1 for the sociodemographic distribution at the city-level in Nigeria and Senegal.

#### Exposure to demand-generation activities

The proportion of men included in our study who reported being exposed to country-specific demand-generation activities is presented in Table 3. Among men surveyed in Kenya, 24 % recalled listening to Tupange’s radio program; 35 % watched Tupange’s television program; and 23 % participated in one of the community events led by Tupange such as group/professional association meetings. About one-half of the men recalled seeing Tupange’s advertisements in print media such as newspaper, magazines, comic books, posters, leaflets, and brochures; while 71 % recalled seeing Tupange’s logo. In all, 85 % of men in the Kenya sample were exposed to at least one of Tupange’s demand-generation activities.

**Table 3:** Proportion of men aged 15–59 exposed to country-specific demand-generation in the three countries

Exposure to programs’ demand-generation activities	Kenya	Nigeria	Senegal
	Tupange (%)	NURHI (%)	ISSU (%)
Listened to any URHI radio programs	24.1	22.9	51.4
Saw any URHI television programs	34.7	52.9	50.5
Participated in any URHI community events	23.2	26.2	8.4
Exposed to any URHI print media materials ^a^	51.9	na	
Exposed to any URHI logos/brands	71.2	29.8	na
Heard/saw any URHI English slogans ^b^	na	33.7	na
Heard/saw any URHI local language slogans ^c^	na	53.9	na
Heard a religious leader speak in favor of family planning	na	na	29.5
Heard at least one URHI radio spot/publicity	na	na	55.8
Exposure to at least one URHI activity	84.8	81.2	79.9
Weighted N	696	2311	1613

All analyses are weighted (cross-city weights were used in Nigeria and Senegal)

*URHI* Urban Reproductive Health Initiative, *NURHI* Nigerian Urban Reproductive Health Initiative, *ISSU* l’Initiative Sénégalaise de Santé Urbaine, *na* not available in country’s program

^a^Tupange program print media includes: newspaper, magazine, comic books, posters, leaflets, and brochures

^b^NURHI’s English slogans: “Get it together”, “know talk go”, “no dulling”

^c^NURHI’s local language (Yoruba and Hausa) slogans: “se o jasi”, “mo ti feto si”, “ki la siri ewa re—ifeto somo bibi lasiri ewa mi”, “ko ku gane, tazaran haihuwa”

In Nigeria, 54 % of men surveyed reported exposure to NURHI’s local language slogans, followed by NURHI’s television programs (53 %); and then NURHI’s English slogans (34 %). About 30 % of men reported seeing at least one of NURHI’s logos and branded products. Only about a quarter had listened to NURHI’s radio program (23 %) or participated in a community event led by NURHI (26 %). In all, 81 % of the men surveyed in Nigeria were exposed to at least one NURHI demand-generation activity. In general, more men in Kaduna compared to Ibadan were exposed to all NURHI demand-generation activities [see Additional file 2].

The situation is slightly different in Senegal where the most common program exposure was the ISSU radio activities. Approximately 56 % of surveyed men in Senegal reported hearing at least one ISSU radio advertisement and 51 % reported listening to ISSU’s radio program. More than half (51 %) reported watching ISSU’s television program. About 30 % heard a religious leader speak in favor of FP but only 8 % participated in an ISSU-led community event. Similar to Kenya and Nigeria, about 80 % of men in the three cities in Senegal were exposed to at least one of the program’s demand-generation activities. See Additional file 2 for the city-level variations in Senegal.

#### Modern contraceptive use at midterm

Table 4 presents the proportion of men who reported they or their partners were using a modern contraceptive method and the type of modern method they were using at the time of the survey. Modern contraceptive use among men was highest in Kenya (58 %), followed by Nigeria (43 %) and then Senegal (27 %). The top three modern methods reported by men in Kenya were male condoms (34 %), injections (24 %), and SDM (15 %). Similar results for main methods used were observed in Nigeria; 50 % were using male condoms, 25 % were using injections, while 7 % were using SDM. The top three methods used in Senegal were slightly different compared to Kenya and Nigeria. The most common method reported by men in Senegal was male condoms (54 %); whereas daily pills and injections were the second and third most common methods reported (19 and 13 %, respectively). The proportion of men reporting female sterilization was low in all three countries; it was higher in Kenya (6 %), compared to Nigeria (2 %) and Senegal (1 %). There was no report of male sterilization, diaphragms, and spermicides in the three countries. See Additional file 3 for the proportion of men reporting modern contraceptive method use at the city-level in Nigeria and Senegal.

**Table 4:** Proportion of men aged 15–59 years using modern contraception in in the three countries

Characteristics	Kenya (%)	Nigeria (%)	Senegal (%)
Current modern method use ^a, b^			
Yes	58.0	42.7	26.6
No	42.0	57.3	73.4
Weighted N	696	2311	1613
Type of modern method ^c, a, b^			
Male condom	34.4	49.7	53.9
Male sterilization	0.0	0.0	
Female sterilization	5.6	1.5	1.0
Daily pills	8.1	4.8	19.3
Injections	23.7	25.4	13.0
Implant	8.2	1.5	7.8
Intrauterine device	3.8	5.6	2.8
Female condom	0.0	3.2	0.1
Emergency pills	1.0	0.0	0.3
Diaphragm/gel/foams	0.0	0.0	
Lactational amenorrhea	0.0	1.8	
Standard days method	15.2	6.5	0.5
Weighted N	403	986	429

All analyses are weighted (across-city weights were used in Nigeria and Senegal)

^a^City-level differences in Nigeria statistically significant at *p* < 0.05

^b^City-level differences in Senegal statistically significant at *p* < 0.05

^c^among modern method users

#### Program exposure and modern contraceptive use

The results of the multivariate logistic regression analyses are presented by country in Table 5. Although city-level analyses were conducted in Nigeria and Senegal, results are not presented in Table 5 but are briefly discussed below. We controlled for the men’s sociodemographic characteristics in all the models. As shown in Table 5, men in Kenya who participated in Tupange-led community events had about four times higher odds of using a modern contraceptive method compared to those who did not participate in such events (aOR: 3.70; 95 % CI: 1.97-6.97). The association between modern method use and exposure to Tupange television program was found to be negative and borderline significant (*p* < 0.10). The other demand-generation activities in Kenya were not significantly associated with modern contraceptive method use (*p* > 0.05).

**Table 5:** Regression of modern contraceptive method use on demand-generation activities in the three countries

Exposure to program demand generation activities	Kenya	Nigeria	Senegal
	OR (95 % C.I.)	OR (95 % C.I.)	OR (95 % C.I.)
Listened to any URHI radio programs	1.36 (0.57–3.24)	0.80 (0.58–1.12)	1.41 (0.98–2.04)^Ɨ^
Saw any URHI television programs	0.58 (0.33–1.01)^Ɨ^	0.91 (0.71–1.18)	1.40 (1.03–1.89)*
Participated in any URHI community events	3.70 (1.97–6.97)***	1.19 (0.93–1.53)	1.14 (0.71–1.81)
Exposed to any URHI print media ^a^	1.02 (0.60–1.75)	na	na
Exposed to any URHI logos/brands	1.05 (0.64–1.74)	1.31 (0.94–1.83)	na
Heard/saw any URHI English slogans ^b^	na	1.39 (0.97–2.01)^Ɨ^	na
Heard/saw any URHI local language slogans ^c^	na	0.92 (0.72–1.17)	na
Heard a religious leader speak in favor of family planning	na	na	1.72 (1.25–2.38)**
Heard at least one URHI radio spot/advert on family planning	na	na	1.29 (0.94–1.77)

All analyses are weighted (across-city weights were used in Nigeria and Senegal). Models include all country-specific program exposure variables controlling for the sociodemographic variables, which are respondents’ age, education, marital status, wealth, religion, and city of residence

*OR* Odds Ratio, *95 % C.I.* 95 % Confidence Interval, *URHI* Urban Reproductive Health Initiative, *na* not available in country’s program

^a^Tupange program print media includes: newspaper, magazine, comic books, posters, leaflets, and brochures

^b^NURHI’s English slogans: “Get it together”, “know talk go”, “no dulling”

^c^NURHI’s local language (Yoruba and Hausa) slogans: “se o jasi”, “mo ti feto si”, “ki la siri ewa re—ifeto somo bibi lasiri ewa mi”, “ko ku gane, tazaran haihuwa”

^Ɨ^ *p* < 0.10; *p < 0.05; **p < 0.01; ***p < 0.001

In Nigeria, the only demand-generation activity that showed a borderline (*p* < 0.10) association with men’s modern method use was exposure to NURHI English language slogans (aOR: 1.39; CI: 0.97–2.01). These slogans included phrases like: "Know, Talk, Go," "No dulling," and "Get it together." When these analyses were run by city, the effect of English language slogans was found to be positive and significant in Kaduna (aOR: 2.01; 95 % CI: 1.13–3.57) but not in Ibadan (aOR: 1.00; 95 % CI: 0.63–1.58); see Additional file 4.

In Senegal, three of the five ISSU demand-generation activities were associated with men’s modern method use. Having seen any of ISSU television programs was positively associated with modern method use (aOR: 1.40; 95 % CI: 1.03–1.89). Additionally, having heard a religious leader speak in favor of FP had a positive association with modern method use (aOR: 1.72; 95 % CI: 1.25–2.38). Finally, exposure to an ISSU radio program had a borderline (*p* < 0.10) positive association with modern method use (aOR: 1.41; CI: 0.98–2.04).

We also assessed the association between the intensity of program exposure to the demand-generation activities and use of modern methods in the three countries. The descriptive statistics and logistic regression results are shown in Table 6. The mean number of demand-generation activities men were exposed to was 2.1 (SD: 1.4) with a range of 0–5 in Kenya; 2.2 (SD: 1.6) with a range of 0–6 in Nigeria; and 2.0 (SD: 1.4) with a range of 0–5 in Senegal. In the bivariate logistic regression (Model 1), the intensity of program exposure was positively associated with modern method use in all three countries; however it is only the association in Senegal that is statistically significant at *p* < 0.001; the associations in Nigeria and Kenya were borderline significant at *p* < 0.10. In the multivariate logistic regression (Model 2), however, only the significant positive association observed in Senegal persisted; though the magnitude of the effect was slightly reduced. Specifically, with each additional exposure to a demand-generation activity, there is a 41 % increase in the odds of reporting use of a modern contraceptive method among men in Senegal (aOR: 1.41; 95 % CI: 1.25–1.60).

**Table 6:** Regression of modern contraception on intensity of program exposure in the three countries

	Kenya	Nigeria	Senegal
	Tupange	NURHI	ISSU
Mean number of program activities exposed to (SD; Range)	2.1 (1.4; 0–5)	2.2 (1.6; 0–6)	2.0 (1.4; 0–5)
Model 1: OR (95 % C.I.)	1.20 (0.98–1.48)*	1.08 (0.98–1.19)^Ɨ^	1.49 (1.32–1.69)**
Model 2: OR (95 % C.I.)	1.15 (0.92–1.44)	1.07 (0.98–1.17)	1.41 (1.25–1.60)**

All analyses are weighted (cross-city weights were used in Nigeria and Senegal)

Model 1-unadjusted (bivariate) logistic regression

Model 2-adjusted logistic regression i.e. intensity of program exposure controlling for sociodemographic variables, which include respondents’ age, education, marital status, wealth, religion, and city

*OR* Odds Ratio; *95 % C.I.* 95 % Confidence Interval, *NURHI* Nigerian Urban Reproductive Health Initiative, *ISSU* l’Initiative Sénégalaise de Santé Urbaine

*p < 0.10; **p < 0.001

Many of the control variables were statistically significant and in the expected directions. For example, in Kenya, there were positive associations between modern method use and being married and being Christian (*p* < 0.05); no significant association was observed for the other control variables. In Nigeria, positive associations were observed between modern method use and age, education, and being married; while being Muslim was negatively associated with modern method use (*p* < 0.05). There was no significant association between household wealth status and modern method use in Nigeria (*p* > 0.05). The role of the control variables was slightly different in Senegal as only age and education were not associated with modern method use (*p* < 0.05) while other control variables had no significant association with modern method use (*p* > 0.05).

## Discussion

This study demonstrates that targeted FP demand-generation activities can lead to improvements in men’s reported use of modern contraception. The Urban Reproductive Health Initiative (URHI) program in these three countries undertook comprehensive demand-generation activities that included mass media (radio and television), interpersonal communication (meetings, outreach activities, and engaging religious leaders), and branding of program materials. These activities were differentially related to men’s modern method use in the varying study contexts. In particular, in Kenya, community outreach events were associated with greater modern method use among men but were not found to be significant among men from the other two countries. In Nigeria, exposure to NURHI’s English Language slogans was associated with modern method use particularly in Kaduna; this suggests that interventions may need to differ across cities. Finally, in Senegal, there were significant associations between modern method use and exposure to radio advertisements/programs and to religious leaders speaking favorably about FP.

Differences found across countries may reflect the types of FP messages used on the radio, television, and in interpersonal communication activities employed in the various settings. The NURHI team used formative research to design their multi-pronged demand-generation strategy that used local language and English language slogans and messages [35]. As seen here, these activities were associated with greater use among men in Kaduna (in the North) but not in Ibadan (in the South). This may reflect greater latent demand for modern FP in Kaduna where the use of modern contraceptive methods at baseline was only 20 % among men as compared to 40 % among men in Ibadan [47]. It may also reflect more effective (or targeted) activities in Kaduna that is a more homogenous city as compared to Ibadan that is very heterogeneous and may require a more diverse set of activities. Further, we found a negative association between exposure to **Tupange** television programming and modern method use among men in Kenya; this might reflect the conservative culture of Mombasa and the fact that the media messages were designed for other less conservative urban areas of the country such as Nairobi and Kisumu. Finally, the differences across countries may relate to the program’s focus, or lack thereof, on men. The ISSU program in Senegal had more focus on men as compared to the URHI programs in Nigeria and Kenya.

Our results that show the association between targeted interventions and men’s use of modern FP are similar to the results found for women, but with some notable differences [31]. Overall, exposure to the program activities was in a similar range between men and women and where there were differences, men were slightly higher on each of the items. Multivariate regression findings demonstrate that for women in Senegal, community outreach activities were significantly associated with modern method use whereas for men in Senegal, it was the exposure to religious leaders speaking favorably about FP that was significant. This distinction may reflect different activity spheres among men and women in urban Senegal but both reflect the role of interpersonal activities as a strategy for influencing FP use in the Senegal. Among women in Nigeria, mass media (radio and television) as well as interpersonal community events were associated with use of modern methods whereas for men, exposure to English language slogans (most likely on the radio) was associated with modern method use. These distinctions between men and women may reflect the need to design activities and messages that better reach men in the varying Nigerian city-level contexts. Among women in Kenya, exposure to Tupange print media and the Tupange radio program was associated with modern method use whereas for men, community events were associated with greater use of modern methods. Community events among men may represent activities targeting men in religious settings in Mombasa, a city that is more Muslim than most cities in Kenya.

This study is not without limitations. First, it is important to note that this study is based on cross-sectional samples of men in three African countries and thus cannot control for time-related changes in population characteristics or directly estimate the causal relationship between exposure to the URHI program and modern method use. Likewise, there is a potential recall bias among respondents as we are asking them to recall their exposure to program activities within the past year. Additionally, these results reflect URHI country-specific program exposure among men only after two years of program implementation. The endline evaluation results may show additional changes and associations between these demand-generation activities and modern contraceptive method use given more time for roll-out of activities and increases in program exposure levels across the study sites. Further, the cities included are not meant to reflect all urban areas within each country. They can only represent the contexts under study, especially in Nigeria and Kenya, where the largest urban areas, Lagos and Nairobi respectively, were not included. In addition, men may over- (or under-) report modern contraceptive method use. This may reflect their desire to give socially desirable responses or a true lack of knowledge of their partner’s contraceptive use. There is also a potential for selection bias for exposure to the URHI demand-generation activities. It is possible that men who have a favorable disposition to FP are able to recall seeing, watching, or participating in any of URHI activities compared to those who do not have this favorable disposition. This bias may also be associated with their probability of practicing FP. In light of these limitations, our study findings should be interpreted with caution.

Despite the limitations, this study provides valuable information. The evidence from prior studies on FP program impacts on men’s contraceptive method use is limited at best. The few studies that examined mass media programs targeted at men’s participation in FP decision-making and use showed positive impacts on contraceptive methods, mainly male condom use [23, 48, 38]. Less is known about program impacts on use of a wider range of modern methods by men and their female partners. Our study begins to fill this gap. While we demonstrate some associations between program exposure and modern contraceptive method use, the main message is that FP programs likely need to not simply “include” men but actually target the program activities and messages to men. This may involve undertaking activities in places where men congregate and/or through specific medium that men use (e.g. religious leaders, television, radio, and sporting events). Furthermore, messages and strategies need to engage men directly (rather than indirectly) as clients, partners, and agents of change. This approach will likely encourage men to communicate with their partners about fertility desires and the need for FP, accompany their partners to a health facility for reproductive health issues, and gain better understanding and need for safe reproductive health behaviors. Additional qualitative and quantitative research is needed with men in varying contexts to better understand the roles men play and/or want to play in reproductive health such that programs can develop messages appropriate to the different contexts. For example, messages developed for one city in Nigeria may not be applicable to another city that represents different ethnic, religious, and socio-demographic groups. Additional research is also needed to better understand the dynamics of couples’ contraceptive choices within varying sociocultural settings.

## Conclusion

To conclude, FP programs need to consider the role of men in influencing contraceptive behaviors of women and couples. This study takes a first step by presenting results from the two-year midterm evaluation of the association between exposure to URHI program activities and men’s reported use of modern contraceptive methods in select urban sites of three African countries. Findings from this analysis are important for informing future FP program activities that seek to engage men and bring them to the table as equal partners in contraceptive adoption and continuation. Program activities should be tailored not just by gender but also by geographic context as results from this study indicate some differences by country and city. It is these types of gender comprehensive and context-specific programming that are likely to be the most successful in reducing unintended pregnancy and unmet need in sub-Saharan Africa.

## Additional files

Additional file 1:
**Sociodemographic characteristics of men aged 15–59 years in the three countries.**
Additional file 2:
**Proportion of men aged 15–59 exposed to country-specific demand-generation activities in the three countries.**
Additional file 3:
**Proportion of men aged 15–59 years using modern contraception in in the three countries.**
Additional file 4:
**Regression of modern contraception on program demand generation activities among men aged 15–59, Mombasa Kenya (2012).**

## Abbreviations

aOR: Adjusted Odds Ratio
BCC: Behavior Change Communication
CI: Confidence Interval
FP: Family Planning
HIV: Human Immunodeficiency Virus
ICPD: International Conference on Population Development
ISSU: l’Initiative Sénégalaise de Santé Urbaine
IUD: Intrauterine device
LAM: Lactational amenorrhea
NURHI: Nigerian Urban Reproductive Health Initiative
PSU: Primary sampling units
SDM: Standard days method
URHI: Urban Reproductive Health Initiative
