# Supplementary material for: Exposure to family planning messages and modern contraceptive use among men in urban Kenya, Nigeria, and Senegal: a cross-sectional study
Source: Reprod Health. 2015 Jul 22;12:63. doi: 10.1186/s12978-015-0056-1 (PMC4508879; doi:10.1186/s12978-015-0056-1)
Supplement: Additional file 1: — Sociodemographic characteristics of men aged 15–59 years in the three countries. [file 12978_2015_56_MOESM1_ESM.docx]

Additional file 1: Sociodemographic characteristics of men aged 15-59 years in the three countries

| **Characteristics** | **Kenya (%)** | **Nigeria (%)** | | | **Senegal (%)** | | | |
| --- | --- | --- | --- | --- | --- | --- | --- | --- |
|  | Mombasa | Total | Ibadan | Kaduna | Total | Guédiawaye | Pikine | Mbao |
| Age |  |  |  |  |  |  |  |  |
| 15-24 | 27.5 | 26.8 | 27.6 | 25.9 | 31.8 | 33.2 | 30.2 | 32.0 |
| 25-34 | 34.3 | 30.6 | 29.1 | 32.2 | 31.8 | 35.2 | 28.0 | 32.2 |
| 35-44 | 21.9 | 22.9 | 23.2 | 22.6 | 21.1 | 19.2 | 24.3 | 19.8 |
| 45+ | 16.3 | 19.7 | 20.1 | 19.3 | 15.3 | 12.4 | 17.5 | 16.0 |
| Education |  |  |  |  |  |  |  |  |
| Primary or less | 42.0 | 14.9 | 16.5 | 13.2 | 55.7 | 43.9 | 64.9 | 57.8 |
| Secondary | 37.2 | 53.5 | 56.8 | 49.7 | 36.1 | 46.9 | 28.0 | 33.9 |
| Higher | 20.7 | 31.6 | 26.7 | 37.1 | 8.2 | 9.2 | 7.1 | 8.3 |
| Marital status |  |  |  |  |  |  |  |  |
| Single/divorced/widowed | 39.7 | 42.6 | 41.0 | 44.4 | 56.3 | 60.7 | 52.5 | 56.0 |
| Married/living together | 60.3 | 57.4 | 59.0 | 55.6 | 43.7 | 39.3 | 47.5 | 44.0 |
| Religion |  |  |  |  |  |  |  |  |
| Christian | 64.7 | 47.0 | 49.1 | 44.7 | 5.4 | 4.9 | 4.7 | 6.4 |
| Muslim | 35.3 | 53.0 | 50.9 | 55.3 | 94.6 | 95.1 | 95.3 | 93.6 |
| Wealth index |  |  |  |  |  |  |  |  |
| Poorest | 21.3 | 20.0 | 18.4 | 21.9 | 25.0 | 23.5 | 26.9 | 24.7 |
| Poor | 22.7 | 19.8 | 21.3 | 18.2 | 18.9 | 18.2 | 16.3 | 22.0 |
| Middle | 21.3 | 20.0 | 20.4 | 19.4 | 19.0 | 16.5 | 18.5 | 21.6 |
| Rich | 17.7 | 20.5 | 21.0 | 19.9 | 18.3 | 19.7 | 17.1 | 18.3 |
| Richest | 17.0 | 19.7 | 18.9 | 20.6 | 18.8 | 22.1 | 21.2 | 13.4 |
| **Weighted N** | **696** | **2311** | **1211** | **1100** | **1613** | **517** | **532** | **564** |
| All analyses are weighted (cross-city weights were used in Nigeria and Senegal). | | | | | | | | |
