# Supplementary material for: Exposure to family planning messages and modern contraceptive use among men in urban Kenya, Nigeria, and Senegal: a cross-sectional study
Source: Reprod Health. 2015 Jul 22;12:63. doi: 10.1186/s12978-015-0056-1 (PMC4508879; doi:10.1186/s12978-015-0056-1)
Supplement: Additional file 2: — Proportion of men aged 15–59 exposed to country-specific demand-generation activities in the three countries. [file 12978_2015_56_MOESM2_ESM.docx]

**Additional file 2**: Proportion of men aged 15-59 exposed to country-specific demand-generation activities in the three countries

| **Exposure to demand-generation activities** | **Kenya: Tupange (%)** | **Nigeria: NURHI (%)** | | | **Senegal: ISSU (%)** | | | |
| --- | --- | --- | --- | --- | --- | --- | --- | --- |
|  | Mombasa | Total | Ibadan | Kaduna | Total | Guédiawaye | Pikine | Mbao |
| Listened to any URHI radio programs | 24.1 | 22.9 | 11.1 | 35.8 | 51.4 | 52.5 | 55.2 | 46.6 |
| Saw any URHI television programs | 34.7 | 52.9 | 42.4 | 64.5 | 50.5 | 41.7 | 54.8 | 54.6 |
| Participated in any URHI community events | 23.2 | 26.2 | 29.2 | 22.8 | 8.4 | 6.1 | 7.8 | 11.1 |
| Exposed to any URHI print media materials ^a^ | 51.9 | na | na | na | na | na | na | na |
| Exposed to any URHI logos/brands | 71.2 | 29.8 | 23.5 | 36.8 | na | na | na | na |
| Heard/saw any URHI English slogans ^b^ | na | 33.7 | 22.9 | 45.7 | na | na | na | na |
| Heard/saw any URHI local language slogans ^c^ | na | 53.9 | 52.6 | 55.5 | na | na | na | na |
| Heard a religious leader speak in favor of FP | na | na | na | na | 29.5 | 20.3 | 37.1 | 30.9 |
| Heard at least one URHI radio spot/publicity | na | na | na | na | 55.8 | 68.1 | 50.7 | 49.2 |
| Exposure to at least one URHI activity | 84.8 | 81.2 | 71.4 | 91.9 | 79.9 | 82.4 | 84.0 | 73.8 |
| **Weighted N** | **696** | **2311** | **1211** | **1100** | **1613** | **517** | **532** | **564** |
| All analyses are weighted (cross-city weights were used in Nigeria and Senegal); na: not available in country’s program.  FP: Family Planning; URHI: Urban Reproductive Health Initiative; NURHI: Nigerian Urban Reproductive Health Initiative; ISSU: l’Initiative Sénégalaise de Santé Urbaine  a Tupange program print media includes: newspaper, magazine, comic books, posters, leaflets, and brochures.  b NURHI’s English slogans: “Get it together”, “know talk go”, “no dulling”.  c NURHI’s local language (Yoruba and Hausa) slogans: “se o jasi”, “mo ti feto si”, “ki la siri ewa re – ifeto somo bibi lasiri ewa mi”, “ko ku gane, tazaran haihuwa”. | | | | | | | | |
