# Supplementary material for: Exposure to family planning messages and modern contraceptive use among men in urban Kenya, Nigeria, and Senegal: a cross-sectional study
Source: Reprod Health. 2015 Jul 22;12:63. doi: 10.1186/s12978-015-0056-1 (PMC4508879; doi:10.1186/s12978-015-0056-1)
Supplement: Additional file 3: — Proportion of men aged 15–59 years using modern contraception in in the three countries. [file 12978_2015_56_MOESM3_ESM.docx]

**Additional file 3**: Proportion of men aged 15-59 years using modern contraception in in the three countries

| **Characteristics** | **Kenya (%)** | **Nigeria (%)** | | | **Senegal (%)** | | | |
| --- | --- | --- | --- | --- | --- | --- | --- | --- |
|  | Mombasa | Total | Ibadan | Kaduna | Total | Guédiawaye | Pikine | Mbao |
| Current modern method use ^ψ, β^ |  |  |  |  |  |  |  |  |
| Yes | 58.0 | 42.7 | 50.8 | 33.8 | 26.6 | 24.1 | 23.6 | 31.7 |
| No | 42.0 | 57.3 | 49.2 | 66.2 | 73.4 | 75.9 | 76.4 | 68.3 |
| **Weighted N** | **696** | **2311** | **1211** | **1100** | **1613** | **517** | **532** | **564** |
| Type of modern method ^a, ψ, β^ |  |  |  |  |  |  |  |  |
| Male condom | 34.4 | 49.7 | 60.2 | 32.4 | 53.9 | 45.3 | 55.0 | 59.2 |
| Male sterilization | 0.0 | 0.0 | 0.0 | 0.0 | 0.0 | 0.0 | 0.0 | 0.0 |
| Female sterilization | 5.6 | 1.5 | 0.5 | 3.1 | 1.0 | 0.0 | 3.4 | 0.0 |
| Daily pills | 8.1 | 4.8 | 3.5 | 6.9 | 19.3 | 20.3 | 11.8 | 23.8 |
| Injections | 23.7 | 25.4 | 20.6 | 33.2 | 13.0 | 12.1 | 21.5 | 7.7 |
| Implant | 8.2 | 1.5 | 0.9 | 2.5 | 7.8 | 10.9 | 4.8 | 7.7 |
| Intrauterine device | 3.8 | 5.6 | 6.3 | 4.4 | 2.8 | 5.6 | 2.9 | 0.9 |
| Female condom | 0.0 | 3.2 | 4.0 | 1.8 | 0.1 | 0.4 | 0.0 | 0.0 |
| Emergency pills | 1.0 | 0.0 | 0.0 | 0.0 | 0.3 | 0.0 | 0.0 | 0.7 |
| Diaphragm/gel/foams | 0.0 | 0.0 | 0.0 | 0.0 | 0.0 | 0.0 | 0.0 | 0.0 |
| Lactational amenorrhea | 0.0 | 1.8 | 1.3 | 2.8 | 1.3 | 4.4 | 0.0 | 0.0 |
| Standard days method | 15.2 | 6.5 | 2.7 | 12.9 | 0.5 | 1.0 | 0.6 | 0.0 |
| **Weighted N** | **403** | **986** | **615** | **371** | **429** | **124** | **126** | **179** |
| All analyses are weighted (across-city weights were used in Nigeria and Senegal)  Ψ City-level differences in Nigeria statistically significant at p<0.05; β City-level differences in Senegal statistically significant at p<0.05; a among modern method users | | | | | | | | |
