# Supplementary material for: Exposure to family planning messages and modern contraceptive use among men in urban Kenya, Nigeria, and Senegal: a cross-sectional study
Source: Reprod Health. 2015 Jul 22;12:63. doi: 10.1186/s12978-015-0056-1 (PMC4508879; doi:10.1186/s12978-015-0056-1)
Supplement: Additional file 4: — Regression of modern contraception on program demand generation activities among men aged 15–59, Mombasa Kenya (2012). [file 12978_2015_56_MOESM4_ESM.docx]

**Additional File 4**

Regression of modern contraception on program demand generation activities among men aged 15-59, Mombasa Kenya (2012)

|  | **OR (95% C.I.)** |
| --- | --- |
| **Exposure to program demand generation activities** |  |
| Listened to any URHI radio programs | 1.36 (0.57 – 3.24) |
| Saw any URHI television programs | 0.58 (0.33 – 1.01)^Ɨ^ |
| Participated in any URHI community events | 3.70 (1.97 – 6.97)*** |
| Exposed to any URHI print media materials ^a^ | 1.02 (0.60 – 1.75) |
| Exposed to any URHI logos/brands | 1.05 (0.64 – 1.74) |
| Model includes adjusted logistic regression i.e. all Tupange program exposure variables + all sociodemographic variables i.e. respondents’ age, education, marital status, wealth, religion (ORs not shown); Ɨ p<0.10; *p<0.05; **p<0.01; ***p<0.001; All analyses are weighted | |

Regression of modern contraception on program demand generation activities among men aged 15-59, Nigeria (2012)

|  | **Total** | **Ibadan** | **Kaduna** |
| --- | --- | --- | --- |
|  | **OR (95% C.I.)** | **OR (95% C.I.)** | **OR (95% C.I.)** |
| **Exposure to program demand generation activities** |  |  |  |
| Listened to any URHI radio programs | 0.80 (0.58 – 1.12) | 0.83 (0.50 – 1.36) | 0.75 (0.49 – 1.17) |
| Saw any URHI television programs | 0.91 (0.71 – 1.18) | 0.99 (0.69 – 1.41) | 0.75 (0.50 – 1.11) |
| Participated in any URHI community events | 1.19 (0.93 – 1.53) | 1.08 (0.79 – 1.48) | 1.31 (0.84 – 2.05) |
| Exposed to any URHI logos/brands | 1.31 (0.94 – 1.83) | 1.00 (0.69 – 1.71) | 1.26 (0.79 – 2.01) |
| Heard/saw any URHI English language slogans | 1.39 (0.97 – 2.01)^Ɨ^ | 1.00 (0.63 – 1.58) | 2.01 (1.13 – 3.57)* |
| Heard/saw any URHI local language slogans | 0.92 (0.72 – 1.17) | 1.31 (0.95 – 1.81) | 0.82 (0.54 – 1.25) |
| Model includes adjusted logistic regression i.e. all NURHI program exposure variables + all sociodemographic variables i.e. respondents’ age, education, marital status, wealth, religion (ORs not shown); Ɨ p<0.10; *p<0.05; **p<0.01; ***p<0.001; All analyses are weighted | | | |

Regression of modern contraception on program demand generation activities among men aged 15-59, Senegal (2013)

|  | **Total** | **Guédiawaye** | **Pikine** | **Mbao** |
| --- | --- | --- | --- | --- |
|  | **OR (95% C.I.)** | **OR (95% C.I.)** | **OR (95% C.I.)** | **OR (95% C.I.)** |
| **Exposure to program demand generation activities** |  |  |  |  |
| Listened to any program radio programs | 1.41 (0.98 – 2.04)^Ɨ^ | 1.10 (0.58 – 2.11) | 2.12 (1.16 – 3.88)* | 1.34 (0.72 – 2.49) |
| Saw any program television programs | 1.40 (1.03 – 1.89)* | 1.31 (0.70 – 2.46) | 1.31 (0.72 – 2.38) | 1.33 (0.90 – 1.95) |
| Participated in any program community events | 1.14 (0.71 – 1.81) | 1.77 (0.64 – 4.94) | 0.90 (0.39 – 2.11) | 0.97 (0.45 – 2.09) |
| Heard a religious leader speak in favor of family planning | 1.72 (1.25 – 2.38)** | 0.84 (0.38 – 1.86) | 1.09 (0.59 – 2.02) | 3.41 (2.17 – 5.34)*** |
| Heard at least one program radio spot/advert on FP | 1.29 (0.94 – 1.77) | 1.08 (0.56 – 2.10) | 0.79 (0.49 – 1.29) | 1.91 (1.06 – 3.45)* |
| Model includes adjusted logistic regression i.e. all ISSU program exposure variables + all sociodemographic variables i.e. respondents’ age, education, marital status, wealth, religion (ORs not shown); Ɨ p<0.10; *p<0.05; **p<0.01; ***p<0.001; All analyses are weighted | | | | |
